# Use of cranial CT to identify a new infarct in patients with a transient ischemic attack

**DOI:** 10.1002/brb3.59

**Published:** 2012-07

**Authors:** Mohamed Al-Khaled, Christine Matthis, Thomas F Münte, Jürgen Eggers

**Affiliations:** 1 Department of Neurology, University of LübeckRatzeburger Allee 160, 23538 Lübeck, Germany; 2 Institute of Social Medicine, University of LübeckRatzeburger Allee 160, 23538 Lübeck, Germany

**Keywords:** CCT, epidemiology, infarct, prognosis, stroke, TIA

## Abstract

Research on infarct detection by noncontrast cranial computed tomography (CCT) in patients with transient ischemic attack (TIA) is sparse. However, the aims of this study are to determine the frequency of new infarcts in patients with TIA, to evaluate the independent predictors of infarct detection, and to investigate the association between a new infarct and early short-term risk of stroke during hospitalization. We prospectively evaluated 1533 consecutive patients (mean age, 75.3 ± 11 years; 54% female; mean National Institutes of Health Stroke Scale [NIHSS] score, 1.7 ± 2.9) with TIA who were admitted to hospital within 48 h of symptom onset. A new infarct was detected by CCT in 47 (3.1%) of the 1533 patients. During hospitalization, 17 patients suffered a stroke. Multivariate logistic regression analysis revealed the following independent predictors for infarct detection: NIHSS score ≥10 (odds ratio [OR], 4.8), time to CCT assessment >6 h (OR 2.2), and diabetes (OR 2.3). The evidence of a new infarct was not associated with the risk of stroke after TIA. The frequency of a new infarct in patients with TIA using CCT is low. The use of the CCT tool to predict the stroke risk during hospitalization in patients with TIA is found to be inappropriate. The estimated clinical predictors are easy to use and may help clinicians in the TIA work up.

## Introduction

Current guidelines recommend the use of cranial computed tomography (CCT) as a routine diagnostic procedure in the evaluation of a transient ischemic attack (TIA) (Johnston et al. 2006; ESO 2008; Easton et al. 2009). Although evidence supporting the use of CCT to detect an infarct in patients suffering from a TIA is sparse, CCT is a mandatory part of clinical practice in the management of patients with acute stroke in emergency departments. A 10-year analysis found that 56% of patients suffering from a TIA who presented to an emergency department underwent CCT (Edlow et al. 2006). In a clinical trial by Koudstaal et al., new ischemic lesions were detected by CCT in 13% of TIAs (Koudstaal et al. 1992). A study by Douglas et al. revealed that 70% of patients with a clinical TIA underwent CCT and that 4% of these patients had a new infarct on CCT (Douglas et al. 2003). Douglas et al. hypothesized that CCT findings may predict the short-term risk of stroke during a follow-up period of 90 days after a TIA. The impact of CCT findings on the early short-term risk of stroke after a TIA has not been previously investigated. However, data on the use of CCT in patients with a TIA are also lacking.

The aims of the present study are to determine the frequency of detection of a new infarct by noncontrast CCT in patients with a TIA who present to hospital within 48 h of symptom onset, to evaluate the independent predictors of infarct detection, and to investigate the association between the presence of a new infarct and the short-term risk of stroke during hospitalization.

## Methods

### Patients and design

During a 36-month period (beginning November 2007), 1533 consecutive patients (mean age, 75.3 ± 11 years; 54% female; mean National Institutes of Health Stroke Scale [NIHSS] score 1.7 ± 2.9; median, 1; interquartile range, 2–5) who were suffering from a TIA and were admitted to the hospital within 48 h of symptom onset and underwent CCT as a diagnostic evaluation of etiology were enrolled in our prospective study as part of a benchmarking project. Of the 15 participating sites, two were university departments of neurology, eight were departments of neurology at nonuniversity hospitals, and five were departments of internal medicine at nonuniversity hospitals. A stroke unit was present in 10 of these hospitals. All patients provided written informed consents for their inclusion in this prospective study. Patients who met the following criteria were included in this study: patients with a TIA (in accordance with the definition that was put forth by the World Health Organization) with symptom lasting less than 24 h, patients who were admitted to the hospital within the first 48 h of symptom onset. The exclusion criteria were an admission to hospital after 48 h of symptom onset, a possible seizure, a history of migraine, and age less than 18 years. The documentation and data collection procedures followed a uniform study manual. Baseline characterizations at admission (Table 1)—gender, age, NIHSS score at admission, duration of symptoms, time to assessment, symptoms of TIA, vascular risk factors, and previous history of stroke—were documented and analyzed. The evidence of a new infarct that was related to the presenting symptoms was abstracted from CCT findings in patients’ radiology reports. The CT scans were read by neuroradiologists who were not involved in the study.

**Table 1:** Baseline characteristics of patients with TIA and factors associated with evidence of a new infarct^1^

Baseline characteristics	All patients *N*= 1533	Infarct on CCT *N*= 47	*P*
Age, mean (SD)	75.3 (11)	74.1 (10.7)	0.4
Female sex	827 (54)	29 (62)	0.2
NIHSS at admission, mean (SD)	1.8 (2.9)	3.5 (4.1)	<0.001
NIHSS score =0 at admission	672 (45)	6 (13)	<0.001
NIHSS score ≥10 at admission	123 (8)	15 (31.9)	<0.001
Symptom duration >1 h	605 (39)	41 (87)	<0.001
Time to admission, h			
<3	142 (24)	5 (19)	
3–6	111 (19)	7 (26)	
6–24	80 (13)	2 (7)	0.47
24–48	264 (44)	13 (48)	
Time to assessment >6 h	344 (31)	15 (48)	0.033
Symptoms of TIA			
Paresis	468 (31)	20 (43)	0.055
Aphasia	314 (20.7)	17 (38)	0.003
Dysarthria	314 (20.5)	15 (32)	0.05
Diabetes	353 (23)	18 (38)	0.01
Hypertension	1261 (82)	39 (83)	0.9
Hyperlipidemia	748 (49)	24 (52)	0.7
Atrial fibrillation	389 (25)	17 (36)	0.09
Previous stroke	456 (30)	13 (28)	0.7
Hospital stay, mean (SD) days	6.2 (4)	8.7 (5)	0.001
Stroke during hospitalization	17 (1.1)	0 (0)	0.4

TIA, transient ischemic attack; CCT, cranial computed tomography; NIHSS, National Institutes of Health Stroke Scale; SD, standard deviation; h, hours.

1 Values in number (percentage) unless otherwise indicated.

The CCT was part of the routine diagnostic evaluation of etiology of the stroke-related neurological symptoms in patients presenting with symptoms of cerebrovascular disease including TIA. The most common CT scan was acquired in the orbitomeatal plane from the skull base through the vertex with a 5-mm slice thickness.

Patients who presented to the hospital with a TIA were diagnosed by at least one neurologist in the department of neurology and by one neurologist in the internal hospitals.

In case of development of stroke-related symptoms during hospitalization, all patients underwent an emergent noncontrast CT and received stroke management and treatment.

A medical doctor who was not involved in the treatment of the patients checked the data. The study was approved by the local ethics committee.

### Statistics

We analyzed the data with an SPSS software program (version, PASW Statistics 18). The mean and standard deviation values were calculated to describe the data. We performed a χ^2^-test to determine the correlation between parametric variables, and a *t*-test between nonparametric variables. Logistic regression analyses were carried out to estimate the odds ratio (OR) for the predictors of the infarct. Variables that were significantly associated with the infarct were evaluated in the logistic regression analyses. A *P-*value less than 0.05 was considered significant.

## Results

During the study period, 1533 consecutive patients met the inclusion criteria. The presence of a new infarct was detected by CCT in 47 (3.1%) of the 1533 consecutive patients who met the inclusion criteria. The most common symptoms of TIA were paresis (30.5%), aphasia (20.7%), and dysarthria (20.5%). In 39% of patients, the symptoms of TIA lasted for more than 1 h. The most common vascular risk factor was hypertension (83%), followed by hyperlipidemia (49%). The percentage of patients who were admitted to hospital within 6 h of symptom onset was 43%. The presence of a new infarct (detected by CCT) associated significantly with an increased NIHSS score (*P* < 0.001), a duration of symptoms of greater than 1 h (*P* < 0.001), length between symptom onset and performance of CCT greater than 6 h (*P*= 0.033), the presence of aphasia at admission (*P*= 0.003), and diabetes as a vascular risk factor (*P*= 0.01) (Table 1).

Using a mulitvariate analysis, we identified an NIHSS score greater than or equal to 10 (OR, 4.8; 95% CI, 2.0–11.2; *P* < 0.001), time to CCT assessment greater than 6 h (OR, 2.2; 95% CI, 1.1–4.6; *P*= 0.029), and diabetes (OR, 2.3; 95% CI, 1.1–4.9; *P*= 0.021) as independent predictors for evidence of a new infarct in patients suffering from a TIA (Table 2).

**Table 2:** Predictors of detection of a new infarct on CCT

	OR	95% CI	*P*
NIHSS ≥10	4.8	2–11.2	<0.001
Time to assessment >6 h	2.2	1.1–4.6	0.029
Diabetes	2.3	1.1–4.9	0.021

During a mean hospitalization of 6 days, 17 patients (1.1%) had an ischemic stroke. None of the patients who suffered a stroke during their hospital stay had exhibited a new infarct on their initial CCT scan. We did not find an association between the evidence of a new infarct on a CCT scan on admission and the short-term risk of stroke during hospitalization following a TIA.

## Discussion

We found evidence of a new infarct by CCT in 3.1% of patients with a TIA in our study. This finding was associated with symptoms of TIA lasting more than 1 h, the length to assessment of more than 6 h, the presence of aphasia at admission, and diabetes as a vascular risk factor. In a previous study, the presence of a new infarct was detected by CCT in 4% of patients with a TIA and was associated with the risk of stroke during a period of 90 days after the TIA (Douglas et al. 2003).

In the present study, 17 patients with a TIA (1.1%) suffered a stroke during hospitalization. We also determined that the early short-term risk of stroke was not associated with the evidence of a new infarct on the initial CCT scan. A previous study reported the risk of stroke to be about 4% among patients with a TIA who presented to hospital with a median time of 3 days (Dennis et al. 1990). The low frequency of stroke in the present study may be explained by early admission, hospitalization of patients, a comprehensive and rapid evaluation including all required diagnostic procedures, and early secondary prophylaxis.

To the best of our knowledge, previous studies have not specifically evaluated the predictors of a new infarct on CCT in patients with a TIA.

Other studies have investigated the relationship between cerebral infarction that is detected by CCT and long-term outcome and suggested that evidence of infarct is correlated with an increase in the risk of recurrent stroke and mortality, but the association between stroke recurrence during hospitalization and infarcts evidence in patients with TIA has not been investigated previously (Evas et al. 1991; van Swieten et al. 1992; Gladstone et al. 2004).

Obviously, the sensitivity of CCT to detect infarcts is considerably lower than that of other imaging techniques. For example, Fazekas et al. (Fazekas et al. 1996) detected a new infarct by MRI in 31% of patients with a TIA. Similarly, Prabhakaran et al. (Prabhakaran et al. 2011), using perfusion computed tomography, found perfusion abnormalities in 33.8% of patients with a TIA. Previous research has also shown that the impact of CCT on visualizing cerebral ischemia in patients with a TIA can be improved with CT perfusion imaging that can provide comprehensive information rapidly (Smith et al. 2003). In summary, the CCT is less sensitive than MRI and diffusion-weighted imaging (DWI) in identification of new infarct in patients with TIA. In the present study, almost (96.9%) of patients did not show a new infarct on CCT. Several investigations, using DWI, demonstrated the frequency of abnormalities in patients with TIA from 41% to 68% that suggest that DWI is a preferable technique in verifying infarcts in patients with TIA and affords more precise detection of ischemic lesion compared to conventional CCT (Kidwell et al. 1999; Ay et al. 2002, 2005; Inatomi et al. 2004; Restrepo et al. 2004; Oppenheim et al. 2006).

Our study has several limitations. The present study did not include the long-term outcome after discharge; the CT findings were abstracted from the radiology reports, which may underestimate the incidence of acute infarction seen on initial CT. The data acquisition was obtained from different hospitals (departments of neurology and internal medicine) without common protocol that could impact the study results, especially in the low rate of stroke risk during hospitalization. However, the work up of TIA follows the uniform recommendation of the German Society of Neurology and German Stroke Society. Another limitation was that the study protocol did not include the findings of the duplex sonography of the arteries in the neck and brain.

Despite these limitations and risks (namely, radiation and iodine contrast exposure) that are associated with CCT (Brenner and Hall 2007), CCT is performed more frequently than MRI on a daily basis for various reasons. These reasons include the fast acquisition, economical factors, ability to reliably exclude a hemorrhage, availability, and the ease of interpretation of CCT findings compared with other diagnostic brain imaging techniques.

## Conclusions

The frequency of acute infarct that is detected by noncontrast enhancement CCT in patients with TIA is low and depends on the severity of the TIA symptoms and the assessment time. The use of the CCT tool to predict the stroke risk during hospitalization in patients with TIA is found to be inappropriate.
